# Correction: Happel et al. Cervicovaginal Human Papillomavirus Genomes, Microbiota Composition and Cytokine Concentrations in South African Adolescents. *Viruses* 2023, *15*, 758

**DOI:** 10.3390/v18101140

**Published:** 2026-10-14

**Authors:** Anna-Ursula Happel, Christina Balle, Enock Havyarimana, Bryan Brown, Brandon S. Maust, Colin Feng, Byung H. Yi, Katherine Gill, Linda-Gail Bekker, Jo-Ann S. Passmore, Heather B. Jaspan, Arvind Varsani

**Affiliations:** 1 Department of Pathology, University of Cape Town, Anzio Road, Observatory, Cape Town 7925, South Africa; anna.happel@uct.ac.za (A.-U.H.); frkballe@gmail.com (C.B.); ehavyarimana@gmail.com (E.H.); jo-ann.passmore@uct.ac.za (J.-A.S.P.); 2 Institute of Infectious Disease and Molecular Medicine, University of Cape Town, Anzio Road, Observatory, Cape Town 7925, South Africa; 3 Seattle Children’s Research Institute, 307 Westlake Ave. N, Seattle, WA 98109, USA; bryan.brown@seattlechildrens.org (B.B.); brandon.maust@seattlechildrens.org (B.S.M.); colin.feng@seattlechildrens.org (C.F.); b.hyunyi@gmail.com (B.H.Y.); 4 Department of Pediatrics, University of Washington, 1510 San Juan Road NE, Seattle, WA 98195, USA; 5 Desmond Tutu Health Foundation, 3 Woodlands Road, Woodstock, Cape Town 7915, South Africa; katherine.gill@hiv-research.org.za (K.G.); linda-gail.bekker@hiv-research.org.za (L.-G.B.); 6 National Health Laboratory Service, Observatory, Cape Town 7925, South Africa; 7 NRF-DST Center of Excellence in HIV Prevention, Centre for the AIDS Programme of Research in South Africa, 719 Umbilo Road, Congella, Durban 4013, South Africa; 8 Department of Global Health, University of Washington, 1959 NE Pacific St., Seattle, WA 98195, USA; 9 The Biodesign Center for Fundamental and Applied Microbiomics, Center for Evolution and Medicine and School of Life Sciences, Arizona State University, Tempe, AZ 85287, USA; 10 Structural Biology Research Unit, Department of Integrative Biomedical Sciences, University of Cape Town, Observatory, Cape Town 7925, South Africa

**Error in Table**

In the original publication [1], there was a mistake in Table 2 as published. Table 2 incorrectly listed HPV40 under Alphapapillomavirus species 7 for isolates HPV40_UC094_LW_V3, HPV40_UC137_LW_V2, and HPV40_UC015_LW_V3. HPV40, however, belongs to Alphapapillomavirus species 8. The corrected Table 2 appears below. 

**Text Correction**

There was an error in the original publication. The manuscript contained inconsistent HPV risk classifications for HPV30, HPV53, and HPV69. The affected text has been revised to remove risk descriptors and present the HPV genotypes without classification labels. A correction has been made to Section 3.2, HPV Infection Patterns in South African Adolescents, Paragraph 3.

We next evaluated HPV co-infection patterns using a model that employs combinatorics to determine the probability that the observed frequency of HPV genotype co-occurrence is significantly greater than expected (positive association), significantly less than expected (negative association), or approximately equal to expected (random association) (Figure 3a). Of the 40 HPV genotypes identified in this study, 35 showed positive associations with one or more HPV genotypes. No associations were found between HPV-62, -66, -67, -215, -mSD2 and any other HPV genotype. There was a significant positive co-occurrence between the presence of HPV-16 and twelve other HPV genotypes, including HPV-18, -30, -33, -40, -43, -44, -53, -54, -56, -61, -68, and -69 (all *p* < 0.05) (Figure 3a). In agreement, samples where HPV-16 was present (*n* = 6) had significantly more other HPV types (median 12, interquartile range (IQR) 5–12) compared to samples where HPV-16 was not present (*n* = 35; median 1, IQR 0–2; *p* < 0.0001). HPV-61 and HPV-69 co-occurred in all samples in which the given genotypes were detected (*p* = 0.0008). This was also true for the co-occurrence of HPV-39 and -68 (*p* = 0.0003) and HPV-40 and -53 (*p* = 0.0003). These data suggest that HPV genotypes frequently co-occur and that the co-occurrence between specific HPV genotypes might be more common than for others.

The authors state that the scientific conclusions are unaffected. This correction was approved by the Academic Editor. The original publication has also been updated.

## Figures and Tables

**Table 2:** List of identified complete HPV genomes determined in this study.

Virus Name	Genus	Species	Type	GenBank Accession
HPV6_UC095_LW_V2	*Alphapapillomavirus*	*Alphapapillomavirus 10*	human papillomavirus 6	OP971086
HPV6_UC096_LW_V2	*Alphapapillomavirus*	*Alphapapillomavirus 10*	human papillomavirus 6	OP971088
HPV6_UC084_LW_V1	*Alphapapillomavirus*	*Alphapapillomavirus 10*	human papillomavirus 6	OP971006
HPV6_UC084_LW_V2	*Alphapapillomavirus*	*Alphapapillomavirus 10*	human papillomavirus 6	OP971011
HPV6_UC096_LW_V3	*Alphapapillomavirus*	*Alphapapillomavirus 10*	human papillomavirus 6	OP971026
HPV16_UC022_LW_V2	*Alphapapillomavirus*	*Alphapapillomavirus 9*	human papillomavirus 16	OP971070
HPV16_UC094_LW_V2	*Alphapapillomavirus*	*Alphapapillomavirus 9*	human papillomavirus 16	OP971046
HPV16_UC094_LW_V3	*Alphapapillomavirus*	*Alphapapillomavirus 9*	human papillomavirus 16	OP971080
HPV16_UC137_LW_V2	*Alphapapillomavirus*	*Alphapapillomavirus 9*	human papillomavirus 16	OP971062
HPV16_UC159_LW_V2	*Alphapapillomavirus*	*Alphapapillomavirus 9*	human papillomavirus 16	OP971067
HPV18_UC008_LW_V3	*Alphapapillomavirus*	*Alphapapillomavirus 7*	human papillomavirus 18	OP971042
HPV18_UC094_LW_V2	*Alphapapillomavirus*	*Alphapapillomavirus 7*	human papillomavirus 18	OP971048
HPV18_UC094_LW_V3	*Alphapapillomavirus*	*Alphapapillomavirus 7*	human papillomavirus 18	OP971083
HPV26_UC096_LW_V2	*Alphapapillomavirus*	*Alphapapillomavirus 5*	human papillomavirus 26	OP971091
HPV30_UC159_LW_V2	*Alphapapillomavirus*	*Alphapapillomavirus 6*	human papillomavirus 30	OP971102
HPV30_UC084_LW_V1	*Alphapapillomavirus*	*Alphapapillomavirus 6*	human papillomavirus 30	OP971007
HPV30_UC084_LW_V2	*Alphapapillomavirus*	*Alphapapillomavirus 6*	human papillomavirus 30	OP971013
HPV31_UC095_LW_V2	*Alphapapillomavirus*	*Alphapapillomavirus 9*	human papillomavirus 31	OP970964
HPV33_UC022_LW_V2	*Alphapapillomavirus*	*Alphapapillomavirus 9*	human papillomavirus 33	OP971069
HPV33_UC015_LW_V1	*Alphapapillomavirus*	*Alphapapillomavirus 9*	human papillomavirus 33	OP970997
HPV34_UC094_LW_V2	*Alphapapillomavirus*	*Alphapapillomavirus 11*	human papillomavirus 34	OP971076
HPV34_UC096_LW_V2	*Alphapapillomavirus*	*Alphapapillomavirus 11*	human papillomavirus 34	OP971093
HPV34_UC084_LW_V1	*Alphapapillomavirus*	*Alphapapillomavirus 11*	human papillomavirus 34	OP971009
HPV35_UC062_LW_V2	*Alphapapillomavirus*	*Alphapapillomavirus 9*	human papillomavirus 35	OP971043
HPV35_UC095_LW_V2	*Alphapapillomavirus*	*Alphapapillomavirus 9*	human papillomavirus 35	OP971054
HPV35_UC137_LW_V2	*Alphapapillomavirus*	*Alphapapillomavirus 9*	human papillomavirus 35	OP971063
HPV35_UC145_LW_V1	*Alphapapillomavirus*	*Alphapapillomavirus 9*	human papillomavirus 35	OP971100
HPV35_UC139_LW_V2	*Alphapapillomavirus*	*Alphapapillomavirus 9*	human papillomavirus 35	OP971032
HPV35_UC139_LW_V3	*Alphapapillomavirus*	*Alphapapillomavirus 9*	human papillomavirus 35	OP971034
HPV39_UC094_LW_V2	*Alphapapillomavirus*	*Alphapapillomavirus 7*	human papillomavirus 39	OP971074
HPV39_UC094_LW_V3	*Alphapapillomavirus*	*Alphapapillomavirus 7*	human papillomavirus 39	OP971084
HPV39_UC095_LW_V2	*Alphapapillomavirus*	*Alphapapillomavirus 7*	human papillomavirus 39	OP971087
HPV39_UC096_LW_V1	*Alphapapillomavirus*	*Alphapapillomavirus 7*	human papillomavirus 39	OP971024
HPV40_UC094_LW_V3	*Alphapapillomavirus*	*Alphapapillomavirus 8*	human papillomavirus 40	OP971079
HPV40_UC137_LW_V2	*Alphapapillomavirus*	*Alphapapillomavirus 8*	human papillomavirus 40	OP971061
HPV40_UC015_LW_V3	*Alphapapillomavirus*	*Alphapapillomavirus 8*	human papillomavirus 40	OP970999
HPV42_UC096_LW_V2	*Alphapapillomavirus*	*Alphapapillomavirus 1*	human papillomavirus 42	OP971089
HPV42_UC113_LW_V2	*Alphapapillomavirus*	*Alphapapillomavirus 1*	human papillomavirus 42	OP971098
HPV42_UC096_LW_V3	*Alphapapillomavirus*	*Alphapapillomavirus 1*	human papillomavirus 42	OP971027
HPV43_UC094_LW_V2	*Alphapapillomavirus*	*Alphapapillomavirus 8*	human papillomavirus 43	OP971044
HPV43_UC094_LW_V3	*Alphapapillomavirus*	*Alphapapillomavirus 8*	human papillomavirus 43	OP971078
HPV43_UC139_LW_V1	*Alphapapillomavirus*	*Alphapapillomavirus 8*	human papillomavirus 43	OP971030
HPV44_UC022_LW_V2	*Alphapapillomavirus*	*Alphapapillomavirus 10*	human papillomavirus 44	OP971071
HPV45_UC094_LW_V2	*Alphapapillomavirus*	*Alphapapillomavirus 7*	human papillomavirus 45	OP971072
HPV45_UC094_LW_V3	*Alphapapillomavirus*	*Alphapapillomavirus 7*	human papillomavirus 45	OP971081
HPV45_UC055_LW_V2	*Alphapapillomavirus*	*Alphapapillomavirus 7*	human papillomavirus 45	OP971003
HPV45_UC139_LW_V2	*Alphapapillomavirus*	*Alphapapillomavirus 7*	human papillomavirus 45	OP971033
HPV51_UC096_LW_V2	*Alphapapillomavirus*	*Alphapapillomavirus 5*	human papillomavirus 51	OP971092
HPV51_UC084_LW_V1	*Alphapapillomavirus*	*Alphapapillomavirus 5*	human papillomavirus 51	OP971008
HPV51_UC084_LW_V2	*Alphapapillomavirus*	*Alphapapillomavirus 5*	human papillomavirus 51	OP971014
HPV51_UC164_LW_V1	*Alphapapillomavirus*	*Alphapapillomavirus 5*	human papillomavirus 51	OP971038
HPV52_UC055_LW_V1	*Alphapapillomavirus*	*Alphapapillomavirus 9*	human papillomavirus 52	OP971002
HPV52_UC084_LW_V2	*Alphapapillomavirus*	*Alphapapillomavirus 9*	human papillomavirus 52	OP971012
HPV52_UC164_LW_V1	*Alphapapillomavirus*	*Alphapapillomavirus 9*	human papillomavirus 52	OP971036
HPV52_UC164_LW_V2	*Alphapapillomavirus*	*Alphapapillomavirus 9*	human papillomavirus 52	OP971039
HPV52_UC164_LW_V3	*Alphapapillomavirus*	*Alphapapillomavirus 9*	human papillomavirus 52	OP971041
HPV53_UC022_LW_V3	*Alphapapillomavirus*	*Alphapapillomavirus 6*	human papillomavirus 53	OP970965
HPV53_UC094_LW_V3	*Alphapapillomavirus*	*Alphapapillomavirus 6*	human papillomavirus 53	OP971053
HPV53_UC137_LW_V2	*Alphapapillomavirus*	*Alphapapillomavirus 6*	human papillomavirus 53	OP971099
HPV53_UC139_LW_V1	*Alphapapillomavirus*	*Alphapapillomavirus 6*	human papillomavirus 53	OP971031
HPV54_UC094_LW_V2	*Alphapapillomavirus*	*Alphapapillomavirus 13*	human papillomavirus 54	OP971049
HPV54_UC137_LW_V2	*Alphapapillomavirus*	*Alphapapillomavirus 13*	human papillomavirus 54	OP971064
HPV56_UC094_LW_V2	*Alphapapillomavirus*	*Alphapapillomavirus 6*	human papillomavirus 56	OP971047
HPV56_UC159_LW_V2	*Alphapapillomavirus*	*Alphapapillomavirus 6*	human papillomavirus 56	OP971068
HPV56_UC091_LW_V1	*Alphapapillomavirus*	*Alphapapillomavirus 6*	human papillomavirus 56	OP971017
HPV56_UC091_LW_V2	*Alphapapillomavirus*	*Alphapapillomavirus 6*	human papillomavirus 56	OP971020
HPV58_UC091_LW_V1	*Alphapapillomavirus*	*Alphapapillomavirus 9*	human papillomavirus 58	OP971016
HPV58_UC164_LW_V1	*Alphapapillomavirus*	*Alphapapillomavirus 9*	human papillomavirus 58	OP971037
HPV58_UC164_LW_V2	*Alphapapillomavirus*	*Alphapapillomavirus 9*	human papillomavirus 58	OP971040
HPV61_UC094_LW_V2	*Alphapapillomavirus*	*Alphapapillomavirus 3*	human papillomavirus 61	OP971045
HPV61_UC094_LW_V3	*Alphapapillomavirus*	*Alphapapillomavirus 3*	human papillomavirus 61	OP971077
HPV61_UC137_LW_V2	*Alphapapillomavirus*	*Alphapapillomavirus 3*	human papillomavirus 61	OP971059
HPV62_UC093_LW_V1	*Alphapapillomavirus*	*Alphapapillomavirus 3*	human papillomavirus 62	OP971022
HPV66_UC115_LW_V3	*Alphapapillomavirus*	*Alphapapillomavirus 6*	human papillomavirus 66	OP971029
HPV67_UC094_LW_V2	*Alphapapillomavirus*	*Alphapapillomavirus 9*	human papillomavirus 67	OP971075
HPV67_UC096_LW_V1	*Alphapapillomavirus*	*Alphapapillomavirus 9*	human papillomavirus 67	OP971025
HPV68_UC094_LW_V2	*Alphapapillomavirus*	*Alphapapillomavirus 7*	human papillomavirus 68	OP971073
HPV68_UC094_LW_V3	*Alphapapillomavirus*	*Alphapapillomavirus 7*	human papillomavirus 68	OP971082
HPV69_UC094_LW_V2	*Alphapapillomavirus*	*Alphapapillomavirus 5*	human papillomavirus 69	OP971051
HPV69_UC094_LW_V3	*Alphapapillomavirus*	*Alphapapillomavirus 5*	human papillomavirus 69	OP971085
HPV69_UC137_LW_V2	*Alphapapillomavirus*	*Alphapapillomavirus 5*	human papillomavirus 69	OP971065
HPV73_UC094_LW_V2	*Alphapapillomavirus*	*Alphapapillomavirus 11*	human papillomavirus 73	OP971050
HPV73_UC119_LW_V2	*Alphapapillomavirus*	*Alphapapillomavirus 11*	human papillomavirus 73	OP971057
HPV74_UC096_LW_V2	*Alphapapillomavirus*	*Alphapapillomavirus 10*	human papillomavirus 74	OP971090
HPV74_UC015_LW_V3	*Alphapapillomavirus*	*Alphapapillomavirus 10*	human papillomavirus 74	OP971000
HPV74_UC096_LW_V3	*Alphapapillomavirus*	*Alphapapillomavirus 10*	human papillomavirus 74	OP971028
HPV81_UC113_LW_V2	*Alphapapillomavirus*	*Alphapapillomavirus 3*	human papillomavirus 81	OP971096
HPV81_UC091_LW_V2	*Alphapapillomavirus*	*Alphapapillomavirus 3*	human papillomavirus 81	OP971019
HPV81_UC091_LW_V3	*Alphapapillomavirus*	*Alphapapillomavirus 3*	human papillomavirus 81	OP971021
HPV81_UC137_LW_V2	*Alphapapillomavirus*	*Alphapapillomavirus 3*	human papillomavirus 81	OP970966
HPV81_UC164_LW_V1	*Alphapapillomavirus*	*Alphapapillomavirus 3*	human papillomavirus 81	OP971035
HPV84_UC137_LW_V2	*Alphapapillomavirus*	*Alphapapillomavirus 3*	human papillomavirus 84	OP971060
HPV84_UC015_LW_V3	*Alphapapillomavirus*	*Alphapapillomavirus 3*	human papillomavirus 84	OP970998
HPV87_UC094_LW_V3	*Alphapapillomavirus*	*Alphapapillomavirus 3*	human papillomavirus 87	OP971052
HPV87_UC055_LW_V1	*Alphapapillomavirus*	*Alphapapillomavirus 3*	human papillomavirus 87	OP971001
HPV87_UC062_LW_V3	*Alphapapillomavirus*	*Alphapapillomavirus 3*	human papillomavirus 87	OP971004
HPV89_UC137_LW_V2	*Alphapapillomavirus*	*Alphapapillomavirus 3*	human papillomavirus 89	OP971058
HPV89_UC096_LW_V1	*Alphapapillomavirus*	*Alphapapillomavirus 3*	human papillomavirus 89	OP971023
HPV90_UC096_LW_V2	*Alphapapillomavirus*	*Alphapapillomavirus 14*	human papillomavirus 90	OP971055
HPV90_UC113_LW_V1	*Alphapapillomavirus*	*Alphapapillomavirus 14*	human papillomavirus 90	OP971094
HPV90_UC084_LW_V1	*Alphapapillomavirus*	*Alphapapillomavirus 14*	human papillomavirus 90	OP971005
HPV90_UC084_LW_V2	*Alphapapillomavirus*	*Alphapapillomavirus 14*	human papillomavirus 90	OP971010
HPV91_UC113_LW_V1	*Alphapapillomavirus*	*Alphapapillomavirus 8*	human papillomavirus 91	OP971095
HPV91_UC113_LW_V2	*Alphapapillomavirus*	*Alphapapillomavirus 8*	human papillomavirus 91	OP971097
HPV108_UC137_LW_V2	*Gammapapillomavirus*	*Gammapapillomavirus 6*	human papillomavirus 108	OP971066
HPV108_UC091_LW_V1	*Gammapapillomavirus*	*Gammapapillomavirus 6*	human papillomavirus 108	OP971018
HPV214_UC113_LW_V2	*Gammapapillomavirus*	unclassified	human papillomavirus 214	OP971056
HPV215_UC145_LW_V1	*Gammapapillomavirus*	unclassified	human papillomavirus 215	OP971101
HPV226_UC084_LW_V2	*Gammapapillomavirus*	unclassified	human papillomavirus 226	OP971015
HPVmSD2_UC094_LW_V3	*Gammapapillomavirus*	unclassified	human papillomavirus mSD2	OP970967
