# Supplementary figures and images for: Cervicovaginal Human Papillomavirus Genomes, Microbiota Composition and Cytokine Concentrations in South African Adolescents
Source: Viruses. 2023 Mar 15;15(3):758. doi: 10.3390/v15030758 (PMC10054107; doi:10.3390/v15030758)

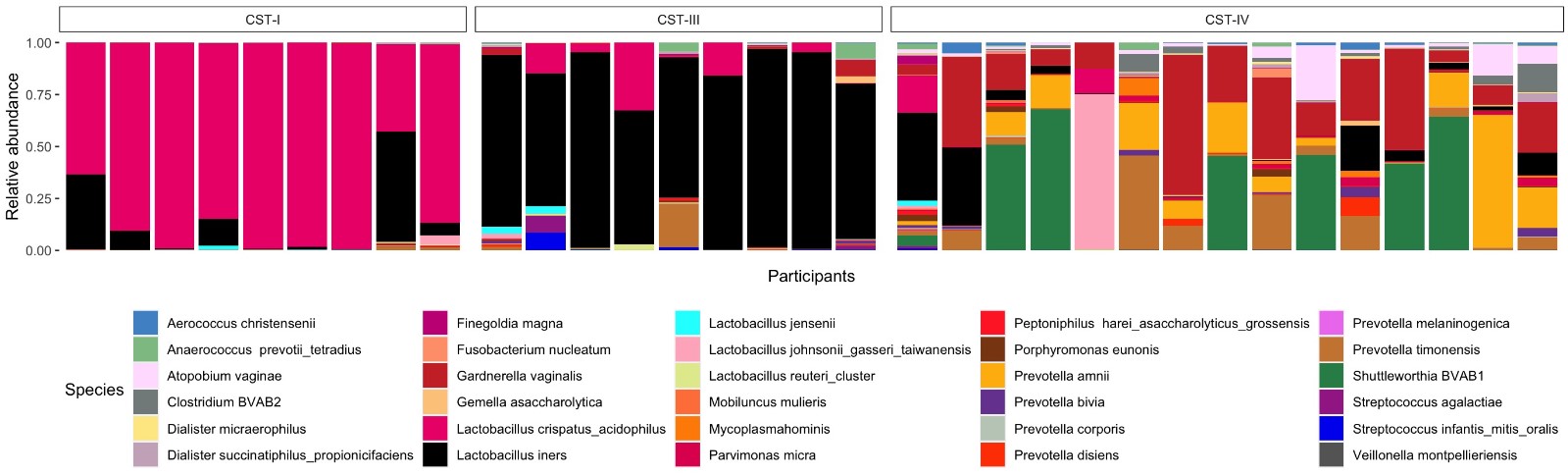

Supplement: Supplementary file 1 [file viruses-15-00758-s001.zip › Figure S1.jpg]
